# Complete genome sequence of *Mediterraneibacter gnavus* strain RI1, isolated from human feces

**DOI:** 10.1128/mra.00863-24

**Published:** 2024-09-30

**Authors:** Ryoha Ichimura, Kazuki Tanaka, Gaku Nakato, Shinji Fukuda, Kazuharu Arakawa

**Affiliations:** 1 Institute for Advanced Biosciences, Keio University, Tsuruoka, Yamagata, Japan; 2 Systems Biology Program, Graduate School of Media and Governance, Keio University, Fujisawa, Kanagawa, Japan; 3 Gut Environmental Design Group, Kanagawa Institute of Industrial Science and Technology, Kawasaki, Japan; 4 Laboratory for Regenerative Microbiology, Juntendo University Graduate School of Medicine, Bunkyo, Tokyo, Japan; 5 Transborder Medical Research Center, Institute of Medicine, University of Tsukuba, Tsukuba, Ibaraki, Japan; 6 Faculty of Environment and Information Studies, Keio University, Fujisawa, Kanagawa, Japan; Rochester Institute of Technology, Rochester, New York, USA

**Keywords:** *Mediterraneibacter gnavus*, complete genome, nanopore sequencing, gut microbiome

## Abstract

We report the complete genome sequence of *Mediterraneibacter gnavus* strain RI1, a Gram-positive anaerobic gut microbe isolated from human feces. The complete circular genome has a genome size of 3.25  Mb, with a G+C content of 42.6%.

## ANNOUNCEMENT

*Mediterraneibacter gnavus* is a Gram-positive anaerobic bacterium that constitutes the gut microbiota and is a common gut bacterium detected in approximately 90% of human intestines (1). Previous studies have reported a positive correlation between *M. gnavus* abundance and symptoms of inflammatory bowel disease, irritable bowel syndrome, and coronary artery disease (2–4).

In this study, we isolated *M. gnavus* strain RI1 from healthy Japanese human feces (Keio SFC Research Ethics Committee approval No. 423). The species of the isolates were determined by a BLAST search of the 16S rRNA gene sequence, which showed 99% identity with *M. gnavus* ATCC 29149. Cultivation of *M. gnavus* strain RI1 was performed overnight in a Gifu Anaerobic Medium (GAM) medium at 37°C in an anaerobic chamber from a glycerol stock. Bacterial genomic DNA was extracted using a NucleoBond high-molecular-weight DNA kit (Macherey-Nagel). The sequencing library for long reads was prepared using a rapid barcoding kit (SQK-RBK114; Oxford Nanopore Technologies), sequenced in a flow cell (FLO-MIN114) with a GridION device (Oxford Nanopore Technologies), base-called (guppy 6.5.7, super accurate mode), and demultiplexed and adapter-trimmed with a GridION X5 version 23.07.12 software (Oxford Nanopore Technologies). The adapter sequences were initially trimmed from the raw reads with Porechop version 0.2.3_seqan2.1.1 (5) and filtered using BBMap (v38.18) (6). The quality of the long reads was assessed using Nanoplot version 1.32.1 (7), resulting in a total of 582,637,653 bp consisting of 7,994 reads of N50 length of 72,164 bp. Reads longer than 50,000 bp with 166× the estimated genome length were used for assembly using Canu version 2.2 (8). The genome was assembled into a single contig and was circularized manually by deleting the overlapping end, following the overlapped coordinates specified by the Canu output. The draft assembly was subsequently error-corrected with three rounds with Medaka version 1.11.3 (Oxford Nanopore Technologies). The completeness of the assembly was assessed with CheckM, and the DDBJ Fast Annotation and Submission Tool was utilized to conduct genome annotation on functional sequences (9, 10). Completeness was 99.38% with a Lachnospiraceae marker lineage. All software was used with their default settings unless otherwise specified.

The assembled genome had a total length of 3,257,867 bp and a G+C content of 42.6%, containing 3,089 protein-coding sequences, 10 rRNAs, and 53 tRNAs. *M. gnavus* genome contains conserved immunoglobulin-binding protein (Ibp) A and IbpB, B cell superantigens that stimulate IgA production (11). In addition, previous studies with metagenomic analysis have reported that *M. gnavus* and its superantigen were present in 42% of individuals from the USA and 43% of individuals from China, but rare among those from Fiji or Tanzania, and were characteristic of different regions (11). Using BLAST+ version 2.2.30 analysis, we found *ibpA* and *ibpB* genes in isolated *M. gnavus* RI1 strain. The availability of the *M. gnavus* RI1 genome resource can potentially contribute to understanding the molecular mechanisms of the disease association.

## Data Availability

The genome sequences reported here were deposited in DDBJ under accession number AP035886, and the raw reads were deposited in the Sequence Read Archive (SRA) under BioProject accession number PRJNA1139382 as SRR29938738 run (http://getentry.ddbj.nig.ac.jp/getentry/na/AP035886/, https://www.ncbi.nlm.nih.gov/bioproject/1139382, https://trace.ncbi.nlm.nih.gov/Traces/?view=run_browser&acc=SRR29938738&display=metadata).
